# University Student Engagement Inventory (USEI): Transcultural Validity Evidence Across Four Continents

**DOI:** 10.3389/fpsyg.2019.02796

**Published:** 2020-01-09

**Authors:** Hugo Assunção, Su-Wei Lin, Pou-Seong Sit, Kwok-Cheung Cheung, Heidi Harju-Luukkainen, Thomas Smith, Benvindo Maloa, Juliana Álvares Duarte Bonini Campos, Ivana Stepanovic Ilic, Giovanna Esposito, Freda Maria Francesca, João Marôco

**Affiliations:** ^1^William James Center for Research, ISPA - University Institute of Psychological, Social and Life Sciences, Lisbon, Portugal; ^2^Department of Education, National University of Tainan, Tainan, Taiwan; ^3^Faculty of Education, University of Macau, Macau, China; ^4^Faculty of Education and Arts, Nord University, Bodø, Norway; ^5^Department of Educational Technology, Research and Assessment, Northern Illinois University, DeKalb, IL, United States; ^6^Universidade Pedagógica de Maputo, Maputo, Mozambique; ^7^School of Pharmaceutical Sciences, São Paulo State University, São Paulo, Brazil; ^8^Department of Psychology, Faculty of Philosophy, University of Belgrade, Belgrade, Serbia; ^9^Department of Humanistic Studies, University of Naples Federico II, Naples, Italy

**Keywords:** student engagement, transcultural invariance, measurment, confirmatory factor analysis, university

## Abstract

Academic engagement describes students’ involvement in academic learning and achievement. This paper reports the psychometric properties of the University Student Engagement Inventory (USEI) with a sample of 3992 university students from nine different countries and regions from Europe, North and South America, Africa, and Asia. The USEI operationalizes a trifactorial conceptualization of academic engagement (behavioral, emotional, and cognitive). Construct validity was assessed by means of confirmatory factor analysis and reliability was assessed using Cronbach’s alpha and McDonald’s omega coefficients. Weak measurement invariance was observed for country/region, while strong measurement invariance was observed for gender and area of graduation. The USEI scores showed predictive validity for dropout intention, self-rated academic performance, and course approval rate while divergent validity with student burnout scores was also evident. Overall, the results indicate that the USEI can produce reliable and valid data on academic engagement of university students across the world.

## Introduction

The concept of engagement emerged in professional and occupational contexts, but has recently been expanded to the educational context as well (Kuh, 2009; Vasalampi et al., 2009; Bresó et al., 2011; Reschly and Christenson, 2012). Student engagement is viewed as a malleable, developing, and multidimensional construct that evolves over time. It can be affected by interventions that enhance positive performance and prevent potential dropout (Appleton et al., 2008). Engaged students invest more in their performance, participate more in school activities, and tend to develop mechanisms to help them persevere and self-regulate their learning processes (Raykov, 2001; Klem and Connell, 2004). Academic engagement is both the cause and consequence of having positive academic and social outcomes (Klem and Connell, 2004; Wonglorsaichon et al., 2014), leading to more satisfaction and self-efficacy (Elmore and Huebner, 2010; Coetzee and Oosthuizen, 2012), and lower incidence of achievement problems and dropout (Fredricks et al., 2004; Gilardi and Guglielmetti, 2011; Reschly and Christenson, 2012).

An early conceptualization of engagement comes from Maslach and Leiter’s (1997) work on the burnout construct. These authors define burnout as the erosion of engagement (Maslach and Leiter, 1997). The burnout syndrome is considered to have three dimensions: emotional exhaustion, depersonalization, and personal accomplishment (Maslach et al., 1996), later generalized to exhaustion, cynicism, and professional efficacy (Schaufeli et al., 1996). Thus, in earlier works engagement was conceptualized as the opposite of burnout and defined as the attribution of meaning and importance to work with feelings of energy, commitment, and accomplishment. When engagement fades, energy turns into exhaustion, involvement turns into cynicism, and efficacy turns into ineffectiveness, leading workers into burnout. In this perspective, people exist in a burnout-engagement continuum in relation to their work (Maslach and Leiter, 1997). However, this conceptualization has a major drawback: people with low levels of burnout are not necessarily engaged in their work. Responding to this critique, a new conceptualization of engagement was proposed by Schaufeli et al. (2002) where three dimensions were considered (vigor, dedication, and absorption), and where engagement was defined as vigor (energy and resilience), absorption (concentration and immersion), and dedication (involvement and enthusiasm). In this view, burnout and engagement, although negatively correlated, are not conceptual opposites. While vigor is the conceptual opposite of exhaustion (activation continuum) and dedication is the opposite of depersonalization/cynicism (identification continuum), absorption and inefficacy are not conceptual opposites (Schaufeli et al., 2002). Absorption is characterized by being “fully concentrated and happily engrossed in one’s work, whereby time passes quickly, and one feels carried away by one’s job.” Based on these nomological considerations, Schaufeli and Bakker (2004) proposed the Utrecht Work Engagement Scale (UWES) to measure engagement. Several authors have since proposed other models that combine behavioral and psychological dimensions (Audas and Douglas Willms, 2001); behavioral, emotional, and cognitive dimensions (Fredricks et al., 2004; Hart et al., 2011); and even a fourth dimension such as academic engagement or agency (Appleton et al., 2008; Reeve and Tseng, 2011; Sinatra et al., 2015). Proposals for the construct dimensionality have ranged from two to eight (learning strategies, academic integration, institutional emphasis, co-curricular activity, diverse interactions, effort, overall relationships, and workload; Lanasa et al., 2009) and higher dimensional models also have been proposed (Martin, 2007).

In this paper, we follow the conceptualization described in Maroco et al. (2016) that expands on the Nystrand and Gamoran (1989) definition of students’ engagement with the North American model (Nystrand and Gamoran, 1989; Fredricks et al., 2004; Maroco et al., 2016). This model has received considerable attention and extensive empirical examination (Janosz et al., 2008; Mo et al., 2008; Archambault et al., 2009; Vasalampi et al., 2009; Bresó et al., 2011; Wang et al., 2011; Tuominen-Soini and Salmela-Aro, 2014; Wang and Fredricks, 2014; Alrashidi et al., 2016; Salmela-Aro and Upadyaya, 2017). Based on this model, Maroco et al. (2016) devised the University Student Engagement Inventory (USEI) which includes behavioral, cognitive, and emotional dimensions of academic engagement with university students. The behavioral dimension is related to positive normative class behaviors (e.g., respecting the social and institutional rules). The cognitive dimension refers to students’ thoughts, perceptions, and strategies related to the acquisition of knowledge or development of competencies to academic activities (e.g., learning approaches). The emotional dimension refers to positive and negative feelings and emotions related to the learning process, class activities, peers, and teachers (Sheppard, 2011; Carter et al., 2012; Maroco et al., 2016). Based on the nomology of the first order engagement constructs, their theoretical closedness as well as the moderate to strong inter-construct correlations, Maroco et al. (2016) proposed a second order factor termed “Engagement.” This second order construct provides an overall measure of the student engagement that unifies the construct (three dimensions, one overall measure), useful for both education psychologists and educators.

Other engagement scales, such as the UWES, have suffered from several criticisms ranging from the construct definitions and dimensionality to its applicability to university students (Lanasa et al., 2009; Wefald and Downey, 2009; Fiorini et al., 2014; Kulikowski, 2017). The USEI was created to measure student engagement in the university context as opposed to the organizational context (Wefald and Downey, 2009; García-Ros et al., 2017) or the elementary student’s context (Fredricks et al., 2011).

Content-related validity evidence based on response processes of the behavioral, cognitive, and emotional as dimensions of academic engagement was evaluated with a focus group of psychologists and university students in the original proposal of Maroco et al. (2016). The USEI has been shown to present appropriate validity, reliability, and measurement invariance across gender and the area of graduation using confirmatory factor analysis (CFA) (Sinval et al., 2018). Although measurement invariance was found across genders and area of studies, no studies so far have analyzed the USEI’s measurement invariance across countries. In this paper, we expect to replicate previous findings by analyzing the USEI’s factorial validity, internal consistency reliability, and convergent and discriminant validity evidence (H1). We also expect the USEI to present measurement invariance across genders, areas of study, and different countries/regions (H2). Finally, we expect that the USEI presents evidence of criterion predictive validity with academic relevant variables such as students’ dropout intention, academic performance, course expectations, course approval rate, and student burnout scores (H3).

## Materials and Methods

### Participants

Minimum sample size for CFA was determined by Monte-Carlo simulation as suggested by Brown (2015) with criteria defined by Muthén and Muthén (2017): (a) Bias of parameters estimates <10%; (b) 95% confidence intervals coverage >91%; and (c) percentage of significant coefficients (power) ≥80%. *Mplus* software (v. 8; Muthén and Muthén, 2017) was used for simulations with the second-order CFA model using factor loadings from the original USEI study (Maroco et al., 2016). A total of 1000 replications employing sample sizes of 100, 200, and 300 were simulated. A minimum sample size of 200 was shown to be enough to attain bias <1% for both parameters and parameters’ standard errors; 99% confidence interval coverage >95%, and minimum power of 90%. However, to ensure that the study sample (which was non-probabilistic) would capture a large amount of the normative population variance we set the sample size at a minimum of 300 students per country/region (i.e., 20 participants per item of the model as suggested by Marôco, 2014).

We collected a sample of 4479 university students (ages ranging from 16 to 70 years; *M* = 23.2; SD = 5.6; Mdn = 21) from Portugal (1067), Brazil (424), Mozambique (413), United Kingdom (314), United States (316), Finland (356), Serbia (409), Macau SAR and Taiwan (762), and Italy (418).

The typical participant was female (60%), pursuing a bachelor’s degree (74%) in human and social sciences (51%) in a public (88%) university (80%), living with their family (54%), which financed their studies (56%) (see Table 1 for further details).

**TABLE 1:** Demographic variables by country.

**Country**	**PT**	**BR**	**MZ**	**UK**	**USA**	**FIN**	**SER**	**TW&MO**	**IT**
Age (mean)	22.9	23.3	26.3	22.6	21.9	26.2	22.0	22.3	23.3
Age (median)	21	22	25	21	20.5	24	22	21	21
Age (SD)	6.7	5.3	6.8	5.3	4.3	7.8	2.2	5.4	6.0
Women (%)	65.3	43.4	62.2	47.1	34.8	62.1	83.9	66.1	77.5
Public school (%)	90	85	97	86	81	94	96	80	90
Human and social sciences (%)	33.4	39.2	71.9	49.0	37.0	56.5	53.3	66.5	30.4
Exact sciences (%)	29.6	38.7	11.4	36.0	50.0	32.3	20.8	22.0	0.0
Biological sciences (%)	9.7	9.2	12.1	8.9	8.2	6.7	4.6	6.3	69.6
Health sciences (%)	27.4	13.0	4.6	6.1	4.7	4.5	15.9	5.1	0.0
1st year (%)	17.7	13.6	39.5	26.3	20.1	15.6	21.3	21.9	21.3
2nd year (%)	18.6	18.1	20.1	20.5	29.4	15.6	21.8	18.4	19.7
3rd year (%)	21.9	20.3	8.7	14.1	36.3	20.8	23.3	28.4	22.2
4th year (%)	15.6	24.1	26.6	22.9	4.3	17.3	28.7	19.1	19.4
5th year (%)	7.0	19.8	3.9	4.7	3.0	28.3	1.3	5.2	8.5
6th year (%)	4.1	1.4	0	4.0	1.3	0.9	1.8	3.7	2.6
7th year (%)	7.3	1.7	0.7	2.0	1.7	0.9	0.8	0.9	2.8
8th year (%)	6.8	0.7	0.5	2.4	2.6	0.3	0	1.6	2.7
9th year (%)	0.6	0.2	0	3.0	1.3	0.3	1.0	0.5	0.7
10th year (%)	0.4	0	0	0	0	0	0	0.3	0.2

*Note: PT - Portugal, BR - Brazil, MZ - Mozambique, UK - The United Kingdom, USA - The United States of America, FIN - Finland, SER - Servia, TW&MO - Taiwan and Macao SAR, IT - Italy.*

### Measuring Instruments

#### University Student Engagement Inventory

The USEI (Maroco et al., 2016) was used to measure student engagement. In the USEI, student engagement is conceptualized as a second-order factor construct that is reflected as behavioral, emotional, and cognitive dimensions. Behavioral engagement is defined as students’ participation in classroom tasks, student conduct, and participation in school-related extracurricular activities. Cognitive engagement is defined as the students’ investment and willingness to exert the necessary efforts for the comprehension and mastering of complex ideas and difficult skills. Emotional engagement is defined as attention to teachers’ instructions, perception of school belonging, and beliefs about the value of schooling. The USEI consists of 15 self-report items, each associated with Likert-type response options ranging from “1-never” to “5-always.” Each of the three first-order factors is composed of five items. The USEI has previously been assessed for factorial validity and reliability (Maroco et al., 2016) and measurement invariance across genders and areas of study (Sinval et al., 2018) but only for Portuguese speaking students. In this study, we used five versions of the scale: Portuguese (for Portugal, Brazil, and Mozambique), English (for the United Kingdom, the United States, and Finland), Serbian (Serbia), Italian (Italia), and simplified Chinese (Macau SAR and Taiwan; see Supplementary Data Sheet 1). The Portuguese and English versions used were the original ones of Maroco et al. (2016). The Serbian, Italian, and simplified Chinese were translated by authors from Maroco et al. (2016) and checked for cross-cultural equivalence.

#### Maslach Burnout Inventory – Student Survey

The Maslach Burnout Inventory – Student Survey (MBI-SSi; Maroco et al., 2014) was used to measure student burnout. Student burnout is conceptualized as a second-order construct reflected on the first-order exhaustion, cynicism, and inefficacy dimensions. The MBI-SSi consists of 15 self-report items rated with a 7-point Likert frequency scale from “0-Never” to “6-Every day.” In its original formulation (Schaufeli et al., 2002), the Efficacy dimension has its items positively worded while Emotional Exhaustion and Cynicism are composed of negatively worded items. Here we use a version of the MBI-SS (MBI-SSi; Maroco et al., 2014) where the items in the Efficacy dimension were negatively worded to give rise to the Inefficacy (INEF) dimension. Four versions of the scale were used in this study: Portuguese (Portugal, Brazil, Mozambique), English (the United Kingdom, United States, and Finland), Serbian (Serbia), and simplified Chinese (Macau and Taiwan).

#### Demographic and Academic-Related Questions

The demographic variables assessed were gender, age, region, household, and financial support. The self-reported academic variables were the name of the degree, area of degree (human and social sciences, exact sciences, biological sciences, and health sciences), type of degree (bachelor’s, master, doctorate), type of school (public/private university), year of school, time of classes, order of preference for the course, self-reported academic performance, dropout intention, total number of classes, and number of failed classes. The class approval rate was calculated by subtracting from one the ratio of the number of failed classes with the number of total classes the student has attended. Five versions of the demographic and academic-related questions were used in this study: Portuguese (Portugal, Brazil, Mozambique), English (the United Kingdom, United States, and Finland), Serbian (Serbia), Italian (Italia), and simplified Chinese (Macau SAR and Taiwan).

### Procedures

An online questionnaire containing two scales measuring student engagement using USEI (Maroco et al., 2016) and student burnout using the MBI (MBI-SSi; Maroco et al., 2014) was created using the Qualtrics platform. The order of appearance of the two scales was randomized between participants. At the end of the questionnaire, participants answered a series of demographic and academic-related questions. The survey was designed to take 15 min to complete. The content, objectives, duration, risks, data policy, ethics approval, and contacts were provided at the start of the questionnaire. Informed consent was required to participate as well as confirmation of enrollment in a higher education institution. To move forward in the questionnaire all answers were mandatory. Only completed questionnaires with no missing data were considered for data analysis. At the end of the survey, participants were asked to voluntarily leave a comment about the survey and to provide their e-mail to receive the results of the study if they wanted to. Faculty members and student associations were contacted in each country/region and invited to distribute the survey via e-mail and online social media.

### Data Analysis

#### Descriptive Statistics and Item Sensitivity

Descriptive statistics were obtained using the *skimr* package (v. 1.0.5; McNamara et al., 2018) and the *psych* package (v. 1.8.12; Revelle and Revelle, 2015) for the *R* statistical system (v. 3.5.3; R Core Team, 2013). The minimum, maximum, average, standard deviation, skewness, and kurtosis were calculated, and histograms were created for each item. Absolute skewness and kurtosis values above 7 and 3, respectively, were considered indicative of strong deviations from normality (Finney and DiStefano, 2013) and low item psychometric sensitivity (Marôco, 2014).

#### Confirmatory Factor Analysis

Confirmatory factor analysis was conducted with the *lavaan* package (v. 0.6.4; Rosseel, 2012) to evaluate the psychometric properties of the data gathered with the USEI and MBI. CFA was conducted to verify whether the first- and second-order factor structure presented an adequate fit for the sample data. We used the following goodness-of-fit indices: χ^2^ (Chi-square statistic), comparative fit index (CFI), Tucker–Lewis index (TLI), root mean square error of approximation (RMSEA), and standardized root mean square residual (SRMR). The fit of the model was considered acceptable when CFI and TLI values were >0.90 and RMSEA and SRMR values were <0.06 and <0.08, respectively (Hu and Bentler, 1999; Marôco, 2014).

Although the USEI items are ordinal, because not all response categories were present in all the nine participant countries/regions, it was not possible to use WLSMV estimation to test threshold invariance. However, when the categorical items have at least five categories and a normal-shaped distribution, as it was observed for our sample, Pearson correlations estimate well the associations between variables (Bentler, 1988; Marôco, 2014). Thus, CFA and analysis of invariance by means of multigroup CFA were carried out using robust maximum-likelihood (MLR) estimation implemented in *lavaan* to account for the small deviations from normality and overestimation of fit indices. No measurement errors of items were correlated for both the USEI and MBI measurement models.

#### Evidence of Convergent and Discriminant Validity Evidence

To analyze the convergent and discriminant validity evidence, the average variance extracted (AVE; Fornell and Larcker, 1981) and the heterotrait–monotrait (HTMT; Henseler et al., 2015) correlations were calculated using the *semTools* package (v. 0.5.1; Jorgensen et al., 2018). Values of AVE ≥ 0.5 were considered acceptable indicators of convergent validity evidence. For two factors *x* and *y*, when AVE*_*x*_* and AVE*_*y*_* ≥ $r_{x\,y}^{2}$ (Fornell and Larcker criterion), or when HTMT correlation values are <0.7, the two factors show evidence of discriminant validity.

#### Evidence of Reliability

Evidence of reliability was assessed using internal consistency measures with the “SemTools” R package (v. 0.5.1; Jorgensen et al., 2018): the Cronbach’s alpha coefficient (α; Cronbach, 1951), the coefficient omega (ω; McDonald, 2013), and the hierarchical omega coefficient (ω_h_; Green and Yang, 2009; McDonald, 2013; Kelley, 2016) for each factor. Alpha and omega values ≥ 0.7 were satisfactory indicators of internal consistency (Marôco, 2014).

#### Evidence of Measurement Invariance

Measurement invariance was tested for country/region, gender, and area of studies. We created a set of comparisons within a group of seven nested models based on the recommendations of Millsap and Yun-Tein (2004) and Wu and Estabrook (2016) for second-order models. A configural model was created, where factor loadings, item intercepts, regression coefficients (second-order structural loadings), first-order factor intercepts, and second-order factor means were freely estimated between groups. This model served as a baseline for further invariance testing. Four nested models were thereafter created where factor loadings, item intercepts, regression coefficients, factor intercepts, and means were sequentially fixed between groups. Fit indices of the nested models were assessed to probe for invariance. Invariance was assessed using the |ΔCFI| < 0.01 criteria (Cheung and Rensvold, 2002) and the |ΔRMSEA| < 0.01 criterion set by Rutkowski and Svetina (2014) were used. χ^2^ difference tests were not used because the large sample sizes would result in statistical significance even when very little invariance was evident. When first-order factor loadings and regression coefficients were invariant between groups, but intercepts were not invariant, weak or metric invariance was assumed. Metric invariance means that the contribution of each item to the factor remains constant across different groups and, thus, relationships of the constructs to other variables can be compared validly among groups. When factor loadings and intercepts were invariant across groups, strong or scalar invariance was assumed. Scalar invariance enables comparisons between group means (Millsap and Yun-Tein, 2004). When factor loadings, intercepts, and second-order factor loadings were invariant across groups, full measurement invariance was assumed. Analysis of invariance may stop at this level because invariance between residuals is considered too restrictive (Marôco, 2014). To ensure equal contributions to the invariance analysis of all eight countries/regions and obtain model convergence, a random sample of 313 students from each participant country/region was drawn from the original sample. To ensure the equal contribution of all areas of study and achieve convergence in invariance analysis between areas of study, a random sample of 335 students from each area was selected from the original sample.

#### Evidence of Criterion and Concurrent-Related Validity

To assess criterion validity, dropout intention, self-rated academic performance, course approval rate, and student burnout scores were simultaneously regressed on student engagement. Evidence of criterion predictive validity was obtained with MLR or probit regression (for ordinal outcomes) using the *lavaan* package (v. 0.6.4; Rosseel, 2012).

#### Student Engagement Scores

Student engagement scores were estimated using the *lavaan* package (v. 0.6.4; Rosseel, 2012) under the weak (metric) invariance assumption among countries/regions. Engagement, behavioral, emotional, and cognitive factors’ scores were estimated, and the following statistics/plots were generated for each dimension: sample size, mean, standard deviation, quartiles, and histogram.

## Results

### Items’ Distributional Properties

Summary measures, including skewness (sk) and kurtosis (ku), as well as the histogram for each of the USEI items are presented in Table 2. No USEI item showed absolute value of ku and sk indicative of strong deviations from the normal distribution or lack of psychometric sensitivity.

**TABLE 2:** Distributional properties of USEI’s items (R, reversed).

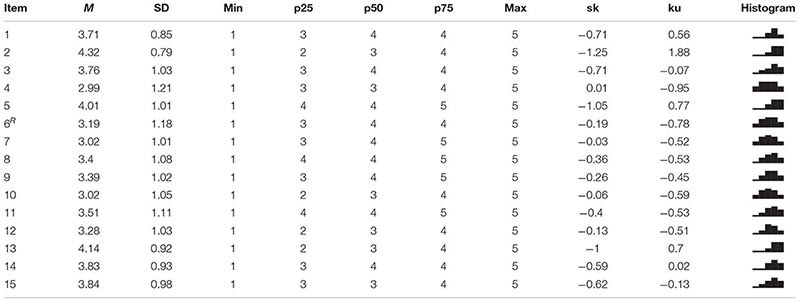

### Factorial Validity Evidence

**FIGURE 1:**
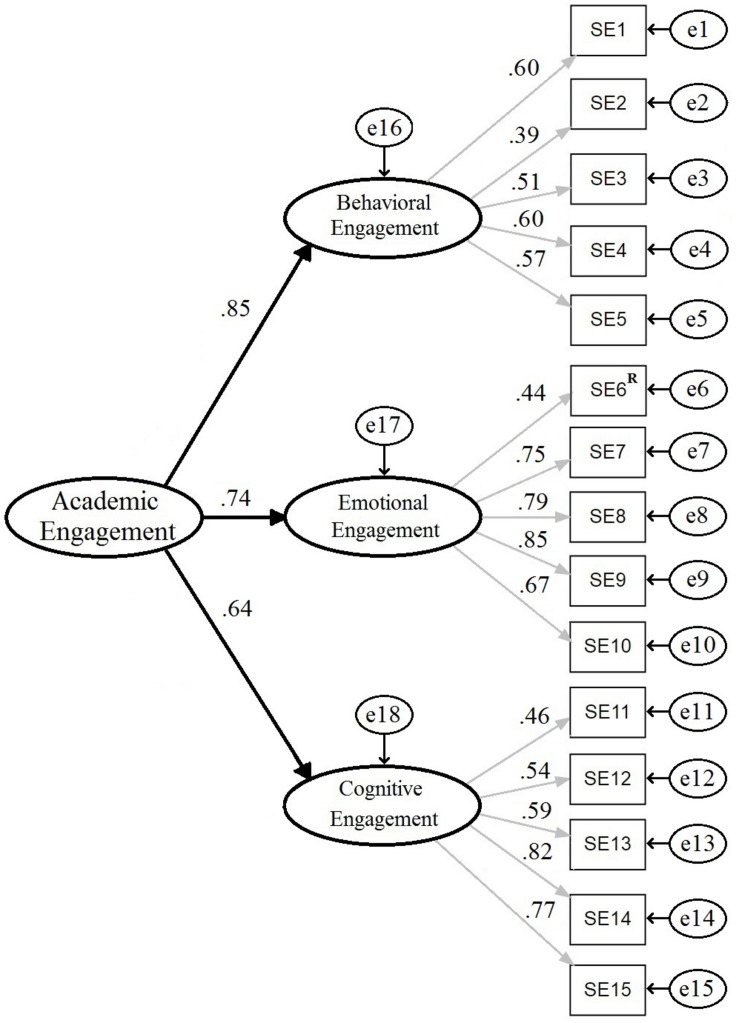
Confirmatory factor analysis of the University Students Engagement Inventory [15 items, R, reversed; χ^2^(87) = 1146.869, CFI = 0.936, TLI = 0.923, RMSEA = 0.052, SRMR = 0.040].

The USEI first-order three-factor model presented an acceptable fit to the data [χ^2^(84) = 751.528, CFI = 0.936, TLI = 0.923, RMSEA = 0.052, SRMR = 0.040). With the addition of a second-order latent variable (Figure 1) goodness of fit indices remained the same. The regression (structural) coefficients for the academic engagement second-order factor model were high for behavioral engagement (γ = 0.85; *p* < 0.001) and emotional engagement (γ = 0.74; *p* < 0.001) and medium for cognitive engagement (γ = 0.64; *p* < 0.001).

### Convergent and Discriminant Validity Evidence

The AVE was acceptable for EE (0.56) and CE (0.49) but low for BE (0.34). Convergent validity evidence was acceptable for the EE and CE factors and poor for the BE factor. The AVE_EE_ was greater than $r_{\mathrm{EE}.\mathrm{CE}}^{2}$ (0.25) and $r_{\text{EE}.\text{BE}}^{2}$ (0.42). The AVE_CE_ was greater than $r_{\text{CE}.\text{EE}}^{2}$ (0.25) and $r_{\text{CE}.\text{BE}}^{2}$ (0.32). The AVE_BE_ was greater than $r_{\text{BE}.\text{CE}}^{2}$ (0.31), but not greater than $r_{\text{BE}.\text{EE}}^{2}$ (0.42) (Table 3). All HTMT inter-construct correlations were below the recommended threshold of 0.70 (HTMT_BE.EE_ = 0.63, HTMT_BE.CE_ = 0.55, and HTMT_EE.CE_ = 0.50). These results altogether show acceptable evidence of convergent- and discriminant-related validity of the USEI dimensions.

**TABLE 3:** Average variance extracted (main diagonal), explained variance (*R*^2^; lower triangular matrix), and HTMT correlations (upper triangular matrix).

**USEI dimension^*a*^**	**BE**	**EE**	**CE**
BE	0.34	0.63	0.55
EE	0.17	0.56	0.50
CE	0.09	0.06	0.49

*^*a*^BE, behavioral engagement; EE, emotional engagement; CE, cognitive engagement.*

### Reliability Evidence

The α values were >0.70 for all factors and >0.8 for the total scale (Table 4). The hierarchical omega statistic for the total scale was high (ω_h_ = 0.88), which gives support to a second-order factor as observed elsewhere (Maroco et al., 2016; Sinval et al., 2018). This result provides evidence of acceptable internal consistency reliability.

**TABLE 4:** Internal consistency reliability of USEI dimensions.

**USEI dimension^*a*^**	**α**	**ω**	**ω hierarchical**
BE	0.71	0.66	0.65
EE	0.85	0.83	0.83
CE	0.81	0.79	0.81
Total	0.87	0.88	0.88

*^*a*^BE, behavioral engagement; EE, emotional engagement; CE, cognitive engagement.*

### Evidence of Measurement Invariance

#### Invariance by Country/Region

To detect whether the second-order latent USEI model holds in different countries/regions, a group of nested models for the nine participating countries/regions was created. Table 5 lists goodness of fit measures for all models (factor loadings, item intercepts, regression coefficients, factor intercepts, and means). Using the Cheung and Rensvold (2002)ΔCFI criterion (|ΔCFI| < 0.01) and the Rutkowski and Svetina (2014)ΔRMSEA criterion (|ΔRMSEA| < 0.01), metric invariance was found between all countries. Following the lack of global scalar invariance, an analysis of invariance was conducted for pairs of countries/participants. Scalar invariance was found between Portugal and Brazil and between the United Kingdom and the United States.

**TABLE 5:** USEI model comparison for country/region invariance.

**Model**	**df**	**χ^2^**	**CFI**	**TLI**	**RMSEA**	**SRMR**	**Δdf**	**Δχ^2†^**	**ΔCFI**	**ΔRMSEA**
Configural	783	1666	0.92	0.904	0.064	0.057				
Loadings	879	1846	0.912	0.905	0.063	0.069	96	181^∗∗∗^	−0.008	−0.001
Intercepts	975	2932	0.824	0.829	0.085	0.084	96	1160^∗∗∗^	−0.088	0.022
Regressions	991	2952	0.823	0.831	0.084	0.087	16	23	−0.001	−0.001
Means	1015	3390	0.786	0.8	0.091	0.108	24	440^∗∗∗^	−0.037	0.007

*^∗∗∗^*p* < 0.001. ^†^Scaled Δχ^2^ test using the Satorra–Bentler correction.*

Information regarding each model’s goodness of fit [χ^2^(df), CFI, TLI, RMSEA, SRMR] and model fitness comparison (Δdf, Δχ^2^, ΔCFI, ΔRMSEA) can be found in Table 5. Information regarding the ΔCFI for each pair of countries can be found in Table 6.

**TABLE 6:** ΔCFI (models with fixed loadings and free intercepts vs. model with fixed loadings plus fixed intercepts) for each pair of countries/regions.

**ΔCFI**	**BR**	**MZ**	**UK**	**USA**	**FIN**	**SER**	**M&T**	**IT**
Portugal	−0.005^∗^	−0.049	−0.043	−0.042	−0.022	−0.026	−0.044	−0.106
Brazil		−0.031	−0.022	−0.021	−0.012	−0.021	−0.035	−0.079
Mozambique	−0.031		−0.062	−0.06	−0.065	−0.081	−0.064	−0.097
United Kingdom	−0.022	−0.062		−0.001^∗^	−0.012	−0.037	−0.052	−0.114
United States	−0.021	−0.06	−0.001^∗^		−0.015	−0.033	−0.04	−0.126
Finland	−0.012	−0.065	−0.012	−0.015		−0.017	−0.042	−0.071
Serbia	−0.021	−0.081	−0.037	−0.033	−0.017		−0.027	−0.108
Macau and Taiwan	−0.035	−0.064	−0.052	−0.04	−0.042	−0.027		−0.131
Italy	−0.079	−0.097	−0.114	−0.126	−0.071	−0.108	−0.131	

*^∗^|ΔCFI| < 0.01. Column names represent the two letter code for the countries found on the first column.*

#### Measurement Invariance by Gender

To detect whether the USEI invariance holds across genders, a group of nested models with indications of equivalence was created. Table 7 lists goodness of fit measures for all models (factor loadings, item intercepts, regression coefficients, factor intercepts, and means). Using the Cheung and Rensvold (2002)ΔCFI criterion (|ΔCFI| < 0.01) and the Rutkowski and Svetina (2014)ΔRMSEA criterion (|ΔRMSEA| < 0.01), scalar measurement invariance was found for gender. Information regarding each model’s goodness of fit [χ^2^(df), CFI, TLI, RMSEA, SRMR] and model’s goodness of fit comparison (Δdf, Δχ^2^, ΔCFI, ΔRMSEA) can be found in Table 7.

**TABLE 7:** USEI model comparison for gender invariance.

**Model**	**df**	**${\text{χ}}_{\text{scaled}}^{2}$**	**CFI**	**TLI**	**RMSEA**	**SRMR**	**Δdf**	**Δχ^2†^**	**ΔCFI**	**ΔRMSEA**
Configural	174	1224	0.939	0.927	0.056	0.041				
Loadings	186	1242	0.939	0.931	0.055	0.041	12	15	0	−0.002
Intercepts	198	1454	0.928	0.924	0.058	0.045	12	237^∗∗∗^	−0.011	0.003
Regressions	200	1463	0.928	0.924	0.057	0.046	2	9^∗^	0	0
Means	203	1488	0.927	0.924	0.057	0.047	3	25^∗∗∗^	−0.001	0

*^∗^*p* < 0.05; ^∗∗∗^*p* < 0.001. ^†^Scaled Δχ^2^ test using the Satorra–Bentler correction.*

#### Measurement Invariance by Area of Study

To detect whether the second-order latent model invariance holds across different areas of study, a group of nested models for the four areas of study (Social Sciences, Exact Sciences, Biological Sciences, and Health Sciences) was created. Table 8 lists the goodness of indicators for all models (factor loadings, item intercepts, regression coefficients, factor intercepts, and means). Using the Rutkowski and Svetina (2014) |ΔRMSEA| < 0.01 criterion, strong measurement invariance was achieved among the four areas of study. Information regarding each model’s goodness of fit [χ^2^(df), CFI, TLI, RMSEA, SRMR] and model’s goodness of fit comparison (Δdf, Δχ^2^, ΔCFI, ΔRMSEA) can be found in Table 8.

**TABLE 8:** USEI model comparison for area invariance.

**Model**	**df**	**χ^2^**	**CFI**	**TLI**	**RMSEA**	**SRMR**	**Δdf**	**Δχ^2†^**	**ΔCFI**	**ΔRMSEA**
Configural	348	982	0.924	0.908	0.063	0.052				
Loadings	384	1027	0.923	0.915	0.061	0.057	36	46.375	−0.001	−0.003
Intercepts	420	1259	0.900	0.900	0.066	0.062	36	247^∗∗∗^	−0.023	0.005
Regressions	426	1262	0.900	0.902	0.065	0.063	6	4.734	0	−0.001
Means	435	1358	0.890	0.894	0.068	0.072	9	100^∗∗∗^	−0.01	0.003

*^∗∗∗^*p* < 0.001. ^†^Scaled Δχ^2^ test using the Satorra–Bentler correction.*

#### MBI Factorial Validity and Internal Consistency Evidence

The first-order three-factor MBI-SSi model presented an adequate fit to the data [χ^2^(87) = 2573.694, CFI = 0.911, TLI = 0.892, RMSEA = 0.084, and SRMR = 0.056]. With the addition of a second-order latent variable, goodness of fit indices remained the same. The regression coefficients for the burnout second-order factor model were high for exhaustion (γ = 0.80; *p* < 0.001), for cynicism (γ = 0.86; *p* < 0.001), and inefficacy (γ = 0.90; *p* < 0.001). The α and ω values were >0.85 for all factors and >0.90 for the total scale. The hierarchical omega for the total scale was high (ω_h_ = 0.943). These results provide evidence of adequate internal consistency reliability.

### Criterion Validity Evidence

The USEI showed predictive criterion-related validity with dropout intention (β = −0.407, *R*^2^ = 0.165, *p* < 0.001), self-reported academic performance (β = 0.533, *R*^2^ = 0.284, *p* < 0.001), course expectations (β = 0.528, *R*^2^ = 0.279, *p* < 0.001), and course approval rate (β = 0.244, *R*^2^ = 0.059, *p* < 0.001). Evidence for divergent validity with the students’ burnout was also observed (*r* = −0.69, *p* < 0.001).

### Student Engagement Scores

Table 9 contains the information of the engagement global and dimensions scores by country/region. Mozambique had mean engagement of 3.37, Italy had a mean of 3.11, the United Kingdom had a mean of 3.05, the United States had a mean of 3.04, Portugal and Finland had means of 3.00, and Serbia had a mean of 2.94. Lowest mean engagement values were observed in Taiwan and Macau (2.83) and Brazil (2.82). However, the reader should refrain from comparing the means between all countries/participants since no scalar invariance was observed (not even partial scalar invariance; data not shown) and thus two mean values that are equal in value can have distinct interpretations. Therefore, comparing mean scores is only valid for the pairs of countries which displayed scalar invariance.

**TABLE 9:** USEI scores (1–5) by country/region.

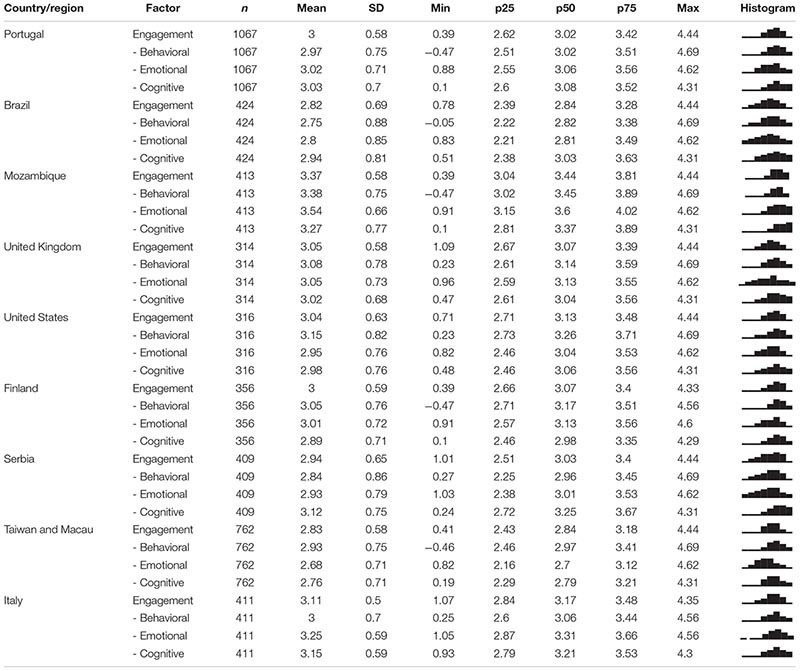

## Discussion

Engagement in university life has proven to be a determinant for learning, academic success, reduce dropout, and promote individual and social well-being (Klem and Connell, 2004; Wonglorsaichon et al., 2014). The measurement of engagement has emerged from the organizational and workplace framework (Schaufeli et al., 2002), but its importance in other activities, like studying, has led to the expansion of the construct and the development of measurement instruments for the school and university context (see, e.g., Appleton et al., 2008; Reeve and Tseng, 2011; Sinatra et al., 2015). In this paper, we report the psychometric properties of engagement data collected with the USEI (Maroco et al., 2016) in higher education systems from nine countries and regions from four continents.

Item sensitivity analysis revealed that the psychometric sensitivity for the 15 items composing the USEI was adequate (Table 1). Further CFA showed that the USEI presented adequate evidence of factorial validity, with goodness-of-fit indices indicating a very good fit of the second-order factorial engagement structure to the data from the nine participant countries/regions. Engagement, as a second-order construct presented high loading values for the first-order behavioral engagement and emotional factors and some-how lower, but still medium for the cognitive factor (Figure 1). Reliability, as evaluated by internal consistency measures, was quite high for the emotional and cognitive factors and medium for the behavioral factor (Table 3). The convergent validity evidence was satisfactory for the cognitive and emotional factors, but low for the behavioral factor. The discriminant validity evidence was appropriate for the emotional and cognitive factors according to the Fornell–Larcker criterion and appropriate for all factors according to the HTMT criterion. These results show that although the three first-order factors of engagement (Cognitive, Emotional, and Behavioral) are strongly correlated, they do measure specific factors of engagement (Table 4). Taken together, these results indicate that the USEI presents adequate internal structure validity with data from higher education systems in countries/regions as diverse as the United States, Taiwan and Macau SAR, Finland, Brazil, Servia, Portugal, Italy, and Mozambique. The three-factor scores of the USEI are valid and reliable measures that can be combined to form a reliable total score of academic engagement. These results are in accordance with previous findings of Portuguese students (Costa et al., 2014; Maroco et al., 2016; Sinval et al., 2018) and with our first hypothesis (H1) with students from nine different countries and regions.

With regards to measurement invariance, strong measurement invariance was found for gender and the four areas (Social Sciences, Exact Sciences, Biological Sciences, and Health Sciences). With regards to measurement invariance between countries/regions, we found evidence of strong measurement invariance between Portugal and Brazil and between the United Kingdom and the United States. The remaining combination of countries achieved only weak measurement invariance (Table 7). These results indicate that the USEI’s mean scores can be directly compared between genders and between areas of study within countries/regions, but not across all accessed countries/regions. This result partially confirms our second hypothesis (H2).

Because weak measurement invariance between participating countries/regions was found, it is possible to compare regression models of USEI scores on criterion variables between different countries/participants. We, therefore, investigated the USEI evidence of predictive criterion validity. The USEI can significantly predict dropout intention, academic performance, course approval rate, and course expectations as well as burnout scores (see, e.g., Maslach and Leiter, 1997). Most strikingly, USEI scores shared almost half of their variance with the burnout scores, and can explain a quarter of the variability of subjective academic performance and dropout intention. These results indicate that the USEI scores are significantly related to other aspects of academic life and can be used to make reasonable predictions about students’ academic success and intention to drop out, therefore confirming our third hypothesis (H3).

The USEI generated data with adequate psychometric characteristics that make it an instrument that produces valid and reliable scores to access student engagement in the university context and its behavioral, emotional, and cognitive dimensions. Other engagement scales that measure engagement, such as the UWES (Schaufeli and Bakker, 2004), have suffered several criticisms ranging from the construct definitions and dimensionality to their application to university students (Lanasa et al., 2009; Wefald and Downey, 2009; Campbell and Cabrera, 2011; Mills et al., 2012). Our results support the adequacy of the USEI to measure student engagement in the university context as opposed to the organizational context (Wefald and Downey, 2009; Upadaya and Salmela-Aro, 2012) or the elementary student’s context (Fredricks et al., 2011). Although psychometric analysis showed adequate psychometric qualities of data gathered with the USEI on a diversity of higher education systems, there is still room for improvement. One issue with the conceptualization of student engagement as behavioral, emotional, and cognitive factors is that the behavioral aspect of student engagement dominates the variance attributed to the USEI’s global score. The high structural coefficient from the second-order engagement to the behavioral first-order factor contrasts with its reduced internal consistency reliability and AVE. When analyzing the behavioral factor item-by-item we found that item 2 (I follow the school’s rules) and item 3 (I usually do my homework on time) somehow produces low factor loadings (0.4 < λ < 0.5), which explain the reduced internal consistency and AVE of this factor. Item 2 also suffered from a ceiling effect, having the highest absolute skewness of all items on the scale. Item 3 refers to homework and may not have the same meaning across different courses and education systems as expressed by some students that commented on the appropriateness of this item for their university experience. The high structural coefficient value of engagement on the behavioral factor shows that this factor can be more important for the global score than the emotional and cognitive factors. If this is the case, conceptualizing sub-types of behavioral engagement could prove to be useful. Future research can assess if the behavioral factor benefits from additional items or item rephrasing to better specify all the different behaviors associated with academic engagement.

The emotional and cognitive factors can also be improved, as items 6 and 11 have low factor loadings (0.4 < λ < 0.5). Item 6 has previously been identified as a problematic item in this scale as it is the only reverse-coded item (Sinval et al., 2018). It can be positively worded as “I feel very accomplished at this school” because reversed items may have reduced sensitivity (as demonstrated by Maroco et al., 2014) for the efficacy dimension of the MBI-SS. Item 11 (“When I read a book, I question myself to make sure I understand the subject I’m reading about”) refers to reading a book and may not have the same relevance across different courses or education systems as many students may use a diverse set of media and often read specific chapters of books. A possible solution to this problem is to modify item 11 so that it is not specific to reading a book (e.g., “When I study, I question myself to make sure I understand the subject I’m studying about”). These hypotheses can lead the way to future research and improvement of the USEI.

### Limitations and Future Research

Because of the cross-sectional nature of the current study causation should not be inferred from the data. It is important to avoid causal interpretations of the results, as that would require that longitudinal and experimental methods be used. Therefore, causal association between the USEI scores and criterion variables are to be taken with caution. Future studies could consider studying these variables using longitudinal and/or experimental methods.

A second limitation of this study is the self-selection and self-report nature of the data collected that may create a self-selection bias and a social desirability bias (e.g., in the course approval rate or the self-rated academic performance). Future research could improve on these methods by gathering data in a more systematic manner from the official students’ records and by using objective criterion variables. Further research should also look at the student engagement predictors and consequences paying special attention to students’ academic performance, health, and well-being.

## Conclusion

This study shows that the USEI with students from nine different countries/regions from Europe, North and South America, Africa, and Asia can be used to collect valid and reliable data on student engagement. It shows the stability of the second-order factor structure observed previously with Portuguese students (Costa et al., 2014; Maroco et al., 2016; Sinval et al., 2018). Metric (weak) invariance was found between countries and scalar (strong) invariance was found between Portugal and Brazil and between the United Kingdom and the United States. Furthermore, the USEI shows strong measurement invariance between genders and between areas of study. USEI scores can confidently be used to predict students’ academic performance and other academic-related variables.

## Data Availability Statement

The datasets generated for this study are available on request to the corresponding author.

## Ethics Statement

The study was properly validated by the ISPA-IU’s Ethics Commission (Process: I/017/02/2019) and the Northern Illinois University International Review Board (decision 1504/2014; FWAA00004025). Informed consent was obtained from all individual participants in the study. All procedures performed in studies involving human participants were in accordance with the ethical standards of the Institutional Ethics Research Committee and with the 1964 Helsinki declaration and its later amendments or comparable ethical standards.

## Author Contributions

HA collected the data, did the data analysis, and wrote the first draft of the manuscript. S-WL, P-SS, K-CC, HH-L, BM, II, TS, JC, GE, and FF collected the data and revised the manuscript critically for important intellectual content. JM planned and coordinated the project, did the data analysis, and co-wrote the manuscript.

## Conflict of Interest

The authors declare that the research was conducted in the absence of any commercial or financial relationships that could be construed as a potential conflict of interest.
