# Supplementary material for: University Student Engagement Inventory (USEI): Transcultural Validity Evidence Across Four Continents
Source: Front Psychol. 2020 Jan 9;10:2796. doi: 10.3389/fpsyg.2019.02796 (PMC6979258; doi:10.3389/fpsyg.2019.02796)
Supplement: Supplementary file 1 [file Data_Sheet_1.docx]

**Annex. USEI versions in Portuguese, English, Serbian, Chinese and Italian**

| **Portuguese** | **English** | **Serbian** | | **Chinese** | **Italian** |
| --- | --- | --- | --- | --- | --- |
| Instructions: | | |  | | |
| Utilizando a escala de resposta, indique com que frequência as seguintes afirmações se aplicam a si: | Using the response scale, indicate how often the following statements apply to you: | Koristeći skalu označite koliko često se ove tvrdnje mogu primeniti na vas: | | 針對下列敘述，指出你有多常這樣做： | Ripensando al suo rapporto con l’università, le chiediamo di leggere le seguenti affermazioni e di attribuire  un punteggio che va da 1 (“mai”) a 5 (“sempre”). |
| Response format: | | | | | |
| 1-Nunca  2- Poucas vezes  3- Algumas vezes  4- A maior parte das vezes  5- Sempre | 1-Never  2- A few times  3- Sometimes  4- Most of the time  5- Always | 1-Nikada  2- Par puta  3- Ponekad  4- Uglavnom  5- Uvek | | 1-從未  2- 少數幾次  3- 有時  4- 多數時候  5- 總是 | 1-Mai  2- Raramente  3- Qualche volta  4- Spesso  5- Sempre |
| USEI Items: | | | | | |
| SE1 - Eu estou atento na aula. | SE1 - I pay attention in class. | SE1 - S pažnjom pratim predavanja. | | SE1 - 上課專心 | SE1 - Presto attenzione in aula. |
| SE2 - Eu sigo as regras da escola. | SE2 - I follow the school’s rules. | SE2 - Poštujem pravila koja važe na fakultetu. | | SE2 - 遵守校規 | SE2 - Seguo le regole dell’università. |
| SE3 - Geralmente faço os trabalhos de casa a tempo e horas. | SE3 - I usually do my homework on time. | SE3 - Uglavnom na vreme uradim domaći ili druge fakultetske | | SE3 - 準時做家課 | SE3 - Di solito faccio i miei compiti in tempo. |
|  |  | obaveze. | |  |  |
| SE4 - Quando tenho dúvidas faço perguntas e envolvo-me nos debates da sala de aula. | SE4 - When I have doubts I ask questions and participate debates in the classroom. | SE4 - Kada mi nešto nije jasno postavljam pitanja i učestvujem u diskusiji na predavanjima. | | SE4 - 疑問時，在課堂發問並參與辯論 | SE4 - Quando ho dei dubbi faccio domande e partecipo ai dibattiti in aula. |
|  |  |  | |  |  |
| SE5 - Geralmente participo ativamente nos trabalhos de  grupo. | SE5 - I usually participate actively in group assignments. | SE5 - Uglavnom aktivno učestvujem u zadacima koji se rade  u grupi. | | SE5 - 積極參與小組作業 | SE5 - Di solito partecipo attivamente ai lavori di gruppo. |
| SE6 - Sinto-me pouco realizado nesta escola. | SE6 - I don’t feel very accomplished at this school. | SE6 - Ne osećam se posebno upsešnim-om na studijama. | | SE6 - 這所學校感覺不太有成就 | SE6 - Mi sento poco realizzato in questa università. |
| SE7 - Sinto-me entusiasmado com o trabalho da escola. | SE7 - I feel excited about the school work. | SE7 - Sa zadovoljstvom radim zadatke ili druge obaveze potrebne za fakultet. | | SE7 - 對學校任務感到興奮 | SE7 - Mi sento entusiasta delle attività universitarie. |
| SE8 - Eu gosto de estar na escola. | SE8 - I like being at school. | SE8 - Volim da budem na fakultetu. | | SE8 - 喜歡待在學校 | SE8 - Mi piace stare all’università. |
| SE9 - Estou interessado no trabalho da escola. | SE9 - I am interested in the school work. | SE9 - Zanimljivo mi je da radim na zadacima i obavezama za fakultet. | | SE9 - 對學校任務感興趣 | SE9 - Sono interessato alle attività universitarie. |
| SE10 - Minha sala de aula é um lugar interessante para estar. | SE10 - My classroom is an interesting place to be. | SE10 - Predavanja na mom fakultetu su interesantna. | | SE10 - 教室是一個有趣的地方 | SE10 - La mia aula è un luogo interessante in cui stare. |
| SE11 - Quando leio um livro, questiono-me a mim próprio para ter certeza que entendo o assunto que estou a ler. | SE11 - When I read a book, I question myself to make sure I understand the subject I’m reading about. | SE11 - Kada čitam knjigu, postavljam sebi pitanja kako bih bio-la siguran-a da sam razumeo-la ono što čitam. | | SE11 - 閱讀書籍時，我會問自己，以確保我理解正在閱讀的主題 | SE11 - Quando leggo un libro, mi interrogo per   essere sicuro/a di capire ciò che sto leggendo. |
| SE12 - Eu converso com outras pessoas fora da escola sobre as matérias que aprendo nas aulas. | SE12 - I talk to people outside the school on matters that I learned in class. | SE12 - Razgovaram sa ljudima van studija o stvarima o kojima sam učio-la na predavanjima. | | SE12 - 和校外的人談論我在課堂上所學到的事 | SE12 - Parlo con persone al di fuori del contesto universitario di contenuti trattati. |
| SE13 - Se não compreendo o significado de uma palavra, eu tento resolver o problema, por exemplo, consultando um dicionário ou perguntando a outra pessoa. | SE13 - If I do not understand the meaning of a word, I try to solve the problem, for example by consulting a dictionary or asking someone else. | SE13 - Ako ne rezumem neku reč, koristim rečnik ili pitam nekoga za značenje te reči. | | SE13 - 若不理解字彙的意義，我會嘗試解決，例如查字典或問其他人 | SE13 -Se non comprendo il significato di una parola, cerco di risolvere il problema, per esempio, consultando un dizionario o chiedendo a qualcun altro. |
| SE14 - Tento integrar os conhecimentos adquiridos para resolver problemas novos. | SE14 - I try to integrate the acquired knowledge in solving new problems. | SE14 - Pokušavam da koristim stečeno znanje u rešavanju novih problema. | | SE14 - 在解決新穎問題時，嘗試整合已習得的知識 | SE14 -Cerco di integrare le conoscenze acquisite per risolvere nuovi problemi. |
| SE15 - Tento integrar as matérias das diferentes disciplinas no meu conhecimento geral. | SE15 - I try to integrate subjects from different disciplines into my general knowledge. | SE15 - Pokušavam da uklopim koncepte iz različitih disciplina u svoj sistem opšteg znanja | | SE15 - 嘗試把來自不同學科的主題融入我的一般知識中 | SE15 - Cerco di integrare i contenuti di diverse discipline con la mia conoscenza generale. |
